# Crystal Structure and Self-Interaction of the Type VI Secretion Tail-Tube Protein from Enteroaggregative *Escherichia coli*

**DOI:** 10.1371/journal.pone.0086918

**Published:** 2014-02-14

**Authors:** Badreddine Douzi, Silvia Spinelli, Stéphanie Blangy, Alain Roussel, Eric Durand, Yannick R. Brunet, Eric Cascales, Christian Cambillau

**Affiliations:** 1 Aix-Marseille Université, Architecture et Fonction des Macromolécules Biologiques, UMR 7257, Campus de Luminy, Case 932, Marseille, France; 2 Centre National de la Recherche Scientifique, Architecture et Fonction des Macromolécules Biologiques, UMR 7257, Campus de Luminy, Case 932, Marseille, France; 3 Laboratoire d’Ingénierie des Systèmes Macromoléculaires, Institut de Microbiologie de la Méditerranée, Centre National de la Recherche Scientifique UMR7255, Aix-Marseille Université, Marseille, France; University of Queensland, Australia

## Abstract

The type VI secretion system (T6SS) is a widespread machine used by bacteria to control their environment and kill or disable bacterial species or eukaryotes through toxin injection. The T6SS comprises a central tube formed of stacked hexamers of hemolysin co-regulated proteins (Hcp) and terminated by a trimeric valine-glycine repeat protein G (VgrG) component, the cell puncturing device. A contractile tail sheath, formed by the TssB and TssC proteins, surrounds this tube. This syringe-like machine has been compared to an inverted phage, as both Hcp and VgrG share structural homology with tail components of *Caudovirales*. Here we solved the crystal structure of a tryptophan-substituted double mutant of Hcp1 from enteroaggregative *Escherichia coli* and compared it to the structures of other Hcps. Interestingly, we observed that the purified Hcp native protein is unable to form tubes *in vitro*. To better understand the rationale for observation, we measured the affinity of Hcp1 hexamers with themselves by surface plasmon resonance. The intra-hexamer interaction is weak, with a K_D_ value of 7.2 µM. However, by engineering double cysteine mutants at defined positions, tubes of Hcp1 gathering up to 15 stacked hexamers formed in oxidative conditions. These results, together with those available in the literature regarding TssB and TssC, suggest that assembly of the T6SS tube differs significantly from that of *Sipho*- or *Myoviridae*.

## Introduction

The type VI secretion system (T6SS) is a widespread versatile machine used by bacteria as a weapon to control their biotope and fight bacterial species or eukaryotes [1]–[3]. The T6SS is composed of a long cytoplasmic tubular structure anchored to the cell envelope by a membrane complex [4]–[7]. The long cytoplasmic structure comprised a number of subunits that share significant structural and functional similarities with bacteriophage tail proteins [8]. It is formed by a tube assembled from stacked hexamers of the hemolysin co-regulated (Hcp) protein [1], [9], [10]. This tube is terminated by a trimer of the valine-glycine repeat protein G (VgrG) [11] that has been hypothesized to pierce the bacterial prey cell wall [6], [12] or the membrane of eukaryotes, allowing toxins delivery into the target cell [13]–[15]. Based on structural homologies, it has been proposed that this Hcp/VgrG assembly resembles the tail and tail tip used by phages to inject their DNA into host cells [1], [11], [16]. Four X-ray structures of Hcp proteins have been determined to date: the Hcp1 and Hcp3 proteins of *Pseudomonas aeruginosa* (1Y12; 3HE1) [1], [17], the EvpC protein of *Edwarsiella tarda* (3EAA) [18] and the unpublished structure of an Hcp protein of *Yersinia pestis* (3V4H). They all have been found to assemble hexameric rings of ∼80 Å diameter harboring a central channel of ∼40 Å, features comparable to those of the bacteriophage major tail proteins (MTP) [7], [19]. The channel diameter is of sufficient size to allow the transit of globular toxin effectors of <25 kDa [20]–[23]. A number of anti-bacterial effectors that have peptidoglycan hydrolase [13] or phospholipase activities [25] have been identified. The genes encoding these effectors are genetically associated with genes that encode immunity proteins that usually bind to and inhibit the activity of the toxin, hence preventing killing between sibling bacteria [13], [26]. It has been recently shown that Hcps are chaperones and transporters of the effectors, as specific Hcp/effector complexes could be observed [24]. It has also been proposed that the Hcp proteins are able to form a tube *in vivo* surrounded by a contractile tail sheath formed by the TssB and TssC components. Fluorescence microscopy experiments using a TssB protein fused to the super folder Green Fluorescent Protein (TssB-sfGFP) have demonstrated that this tail sheath cycles between extended and contracted conformations, suggesting that effector delivery by the T6SS involves a contractile mechanism similar to that of bacteriophages [6], [15], [27]–[29]. Tail sheath contraction occurs in a few tens of second, propelling the internal Hcp tube towards the prey cells [6], [15]. Indeed, recent data have shown that prey cell killing coincides with T6SS sheath contraction [15]. After contraction, the ClpV ATPase is recruited to the contracted tail sheath complex and catalyzes its disassembly, to target the TssB and TssC proteins to degradation or to allow new run of assembly [6], [30].

We recently embarked in an exhaustive structural and functional study of the components that assemble the Sci-1 T6SS of enteroaggregative *E. coli* (EAEC) [31]–[34]. Here, we report the crystal structure of an Hcp1 tryptophane derivative and the characterization of Hcp1 self-interaction and self-assembly.

## Results

### Structure Determination Strategy

**Figure 1:**
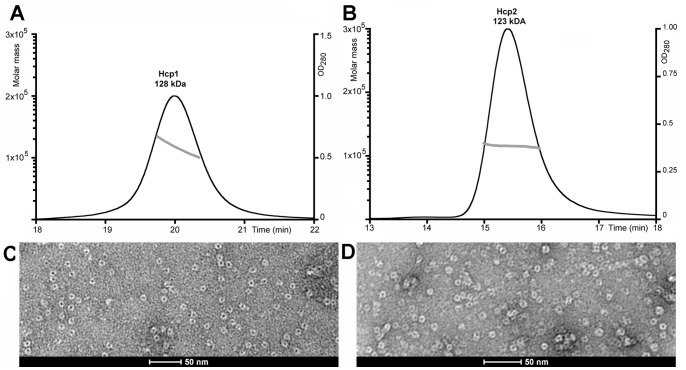
SEC/MALS/RI and electron microscopy analysis of the Hcp1 and Hcp2 proteins. (A, B) SEC/MALS/RI chromatograms of Hcp1 (A) and of Hcp2 (B). The molar mass (left axis, solid line) and the UV_280 nm_ absorbance (right axis, grey line) are plotted as a function of the column elution volume. (C, D) Transmission electron micrographs of negatively stained Hcp1 (C) and Hcp2 (D). Scale bar, 50 nm.

We cloned and produced the Hcp1 protein (accession number: EC042_4529; gene ID: 387609950) using our standard procedures [35]. Hcp1 was purified to homogeneity and was further characterized by biophysical methods. In parallel, the Hcp2 protein, encoded by a second T6SS gene cluster on the EAEC chromosome (accession number EC042_4564; gene ID 387609980), was also produced and purified. MALLS-UV experiments on Hcp1 and Hcp2 revealed that they form particles of 128 kDa and 123 kDa respectively that likely corresponds to hexamers (114 kDa and 111 kDa theoretical weight for Hcp1 and Hcp2 hexamers, respectively) (Fig. 1A, C). Electron microscopy (EM) of negative-stained Hcp1 and Hcp2 further showed that both proteins have a well-defined donut shape (Fig. 1B, D). Both proteins were subjected to crystallization trials, and both crystallized readily. Hcp1 yielded crystals diffracting to 3.5 Å with space group P2 and cell dimensions a = 147.4, b = 85.1, c = 408.5 and ß = 97.3°. Vm calculations revealed that these crystals may contain more than >60 molecules in the asymmetric unit. Structure determination βy molecular replacement failed with this crystal form. Despite tremendous efforts, these crystals could not be improved, and no other crystal forms were obtained. The Hcp2 crystals behaved similarly. We hypothesized that these problems might be due to improper stacking of the hexamers in the crystal. In a recent work, we showed that in absence of the T6SS, the Hcp1 hexamers assemble in head-to-tail, head-to-head and tial-to-tail conformations [10]. Interestingly, substitutions of residues at the hexamer-hexamer interface (at position N93 and S158) by bulky tryptophane residues disrupted tube formation *in vivo* [10]. We therefore introduced the same substitutions to cause unfavourable contacts with the goal to change the crystal packing compared to the native Hcp1 protein. The N93W-S138W double mutant (Hcp1_WW_) crystallized readily and exhibited a good diffraction pattern to 1.69 Å resolution. The structure of EAEC Hcp1 was solved by molecular replacement using the structure of Hcp3 (PDB entry 3HE1) from *P. aeruginosa* as starting model.

### Overall Structure of the Hcp1_WW_ Mutant

**Figure 2:**
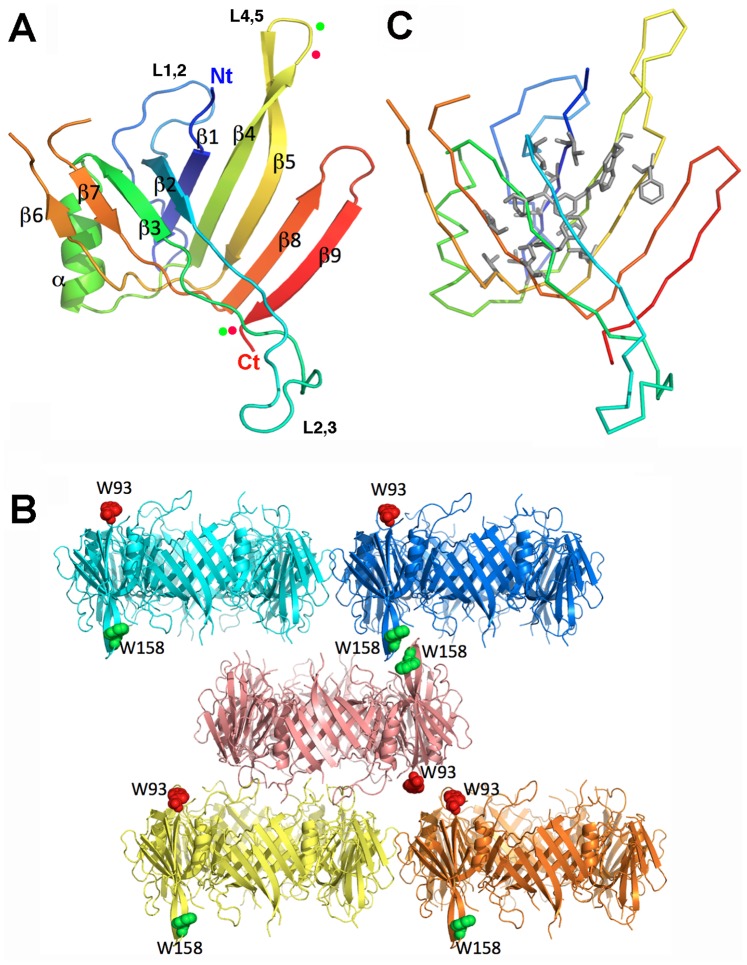
Structure of the Hcp1 N93W/S158W (Hcp1_WW_) protein. (A) Ribbon representation of the Hcp1_WW_ structure. The N- and C- termini are indicated (Nt and Ct respectively). The red dots indicate positions 93 and 158 while green dots indicate the 96 and 158 positions. (B) Carbone α backbone of Hcp1_WW_. The side chains of the residues forming the hydrophobic core are shown in grey. (C) Section of the Hcp1_WW_ crystal packing. 5 Hcp1_WW_ hexameric rings are shown in different colors to aid visualization. The Trp-93 and Trp-158 side chains are represented in red and green spheres respectively.

The structure of Hcp1 was solved and refined as indicated in the material and methods section (Table 1). The electron density map of Hcp1_WW_ was well defined between residues Ala-2 to Val-120 and Ala-129 to Trp-158 (Fig. 2A). The overall Hcp1_WW_ structure revealed a typical Hcp-family fold with two ß-sheets consisting of 4 and 5 ß-strands each and a short α-helix (Fig. 2A). The first β-sheet is formed by strands ß1, ß4, ß5, ß8, ß9 and packs against the second ß-sheet formed by strands ß2, ß3, ß6, ß7 thus forming a ß-barrel fold (Fig. 2A). The well packed interior of this fold is maintained by an hydrophobic core constituted of Val-5, Leu-7 Leu-9 in ß1, Val-32 on ß2, Phe-59, Phe-61 in ß3, Leu-82, Ala-85, Phe-87, Trp-89 in β4, Phe-102, Leu-106 in ß5, Val-111 in the L5–6 loop, Val-135, Leu-137, Tyr-139 in the L7–8 loop and Trp-144 in ß9 (Fig. 2A). The alpha-helix (Ser-67 to Thr-78) is located on one side of the β-barrel and is stabilized by hydrophobic interactions with the ß6 and ß7 strands from the same subunit. In addition, the α-helix is involved in hexameric association due to its hydrophobic interaction with the ß8 and ß9 strands of adjacent subunits (Fig. 2B).

**Table 1:** Data collection and refinement statistics.

DATA COLLECTION	
PDB	4HKH
Source	ESRF ID29
Space group, cell dimensions (Å, °)	C2, 84.2, 145.9, 89.85, ß = 103.4
Resolution limits^a^ (Å)	50.0−**1.69** (1.75−1.69)
Rmeas^a^ (%)	11.0 (77)
Nr. of observations^a^	401232 (40094)
Nr. unique reflections^a^	116804 (11432)
Mean((I)/sd(I))^a^	8.1 (2.0)
Completeness^a^ (%)	99.4 (99.4)
Multiplicity^a^	3.8 (3.7)
**REFINEMENT**	
Resolution^a^ (Å)	25.2−**1.69** (1.79−1.75)
Nr of reflections^a^	116804 (8189)
Atoms : protein, SO_4_, water	7018/55/722
Nr test set reflections	2100
R_work_/R_free_ a (%)	0.181/0.196 (0.23/0.25)
r.m.s.d.bonds (Å)/angles (°)	0.010/1.14
B-wilson/B-average (Å^2^)	20.5/27.7
Ramachandran:preferred/allowed/outliers (%)	96.1/3.3/0.6

a numbers in brackets refer to the highest resolution bin.

The Hcp1_WW_ crystal asymmetric unit contains 6 subunits associated in a hexameric ring. The outside diameter of the donut-shaped hexamer is 80 Å and the inner diameter of its internal channel is 40 Å. The packing of Hcp1_WW_ crystal reveals that Hcp1_WW_ hexamers are not associated in the same organization compared to other Hcp assemblies, which have been described as head-to-tail [1], head-to-head [18] or tail-to-tail [17]. Each Hcp1_WW_ hexamer interacts with two other hexamers on each face, in a frameshift packing of ∼½ hexamer, and in a head-to-head fashion (Fig. 2C). However, this unusual packing was probably induced by the two Trp substitutions, since the Trp-93 or Trp-138 residues of one hexamer interact with a Trp residue of a symmetry related hexamer (Fig. 2C).

### Comparison with other Hcp Structures

**Figure 3:**
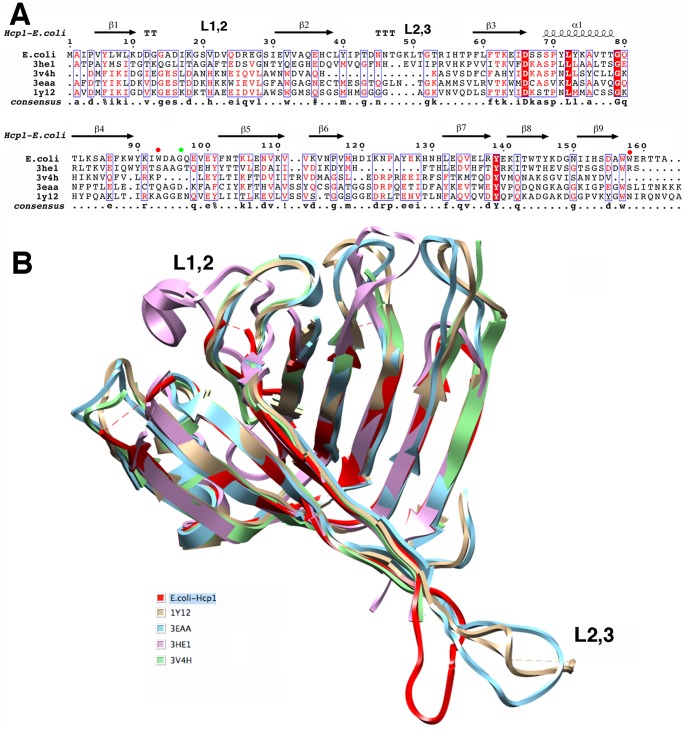
Sequence alignment and structural superimposition of Hcp1_WW_ with other crystallized Hcp proteins. (A) Sequence alignment of the EAEC Hcp1_WW_ protein, Hcp3 from *P. aeruginosa* (3HE1), Hcp from *Y. pestis* (3V4H), EvpC from *E. tarda* (3EAA) and Hcp1 from *P. aeruginosa* (1Y12). Residues targeted in this study are indicated by dots (red for N93, S158 and green for G96). (B) Ribbon representation of the superimposition of Hcp1_WW_ with the indicated Hcp proteins. The color corresponding to each structure is indicated.

To date, four structures of Hcp proteins have been made available (see Table 2). The EAEC Hcp1 protein and the four other Hcps share limited sequence identities ranging from 14% to 40% (Fig. 3A, Table 2). However, the secondary structures are well conserved and the overall tertiary structures of the Hcp proteins are very similar, with rmsd values comprised between 0.7 Å and 1.5 Å, matching the sequence identity order (Table 2). While the backbones of secondary structures match very closely, the main differences occur in the loops, especially the overhang loops L1,2 and L2,3 (Fig. 3B). The *P. aeruginosa* 3HE1 structure is the most different within the L1,2 loop, while the EAEC Hcp1 diverges from the other structures within the L2,3 loop (Fig. 3B).

**Table 2:** Comparison of the five Hcp of known structures.

	4HKH	3V4H	3EAA	3HE1	1Y12
4HKH *E.coli*		**23**	**22**	**36**	**25**
3V4H *Y.pestis*	1.1		**30**	**23**	**40**
3EAA *E. tarda*	1.3	0.9		**17**	**33**
3HE1 *P. aeruginosa PA0263*	1.2	1.3	1.5		**14**
1Y12 *P. aeruginosa PAO1*	1.2	0.7	0.9	1.5	

The sequence identities are in bold (%, above diagonal) and the rmsd values are in italics (in Å, below diagonal).

4HKH: Hcp1 from *E.coli* pathotype EAEC (this work).

3V4H: Hcp from *Yersinia pestis* (unpublished).

3EAA: Hcp from *Edwarsiella tarda* [18].

3HE1: HcpA from *Pseudomonas aeruginosa* PA0263 [17].

1Y12: Hcp from *Pseudomonas aeruginosa* PAO1 [1].

### Hcp1 Self-interaction Studies by Surface Plasmon Resonance

**Figure 4:**
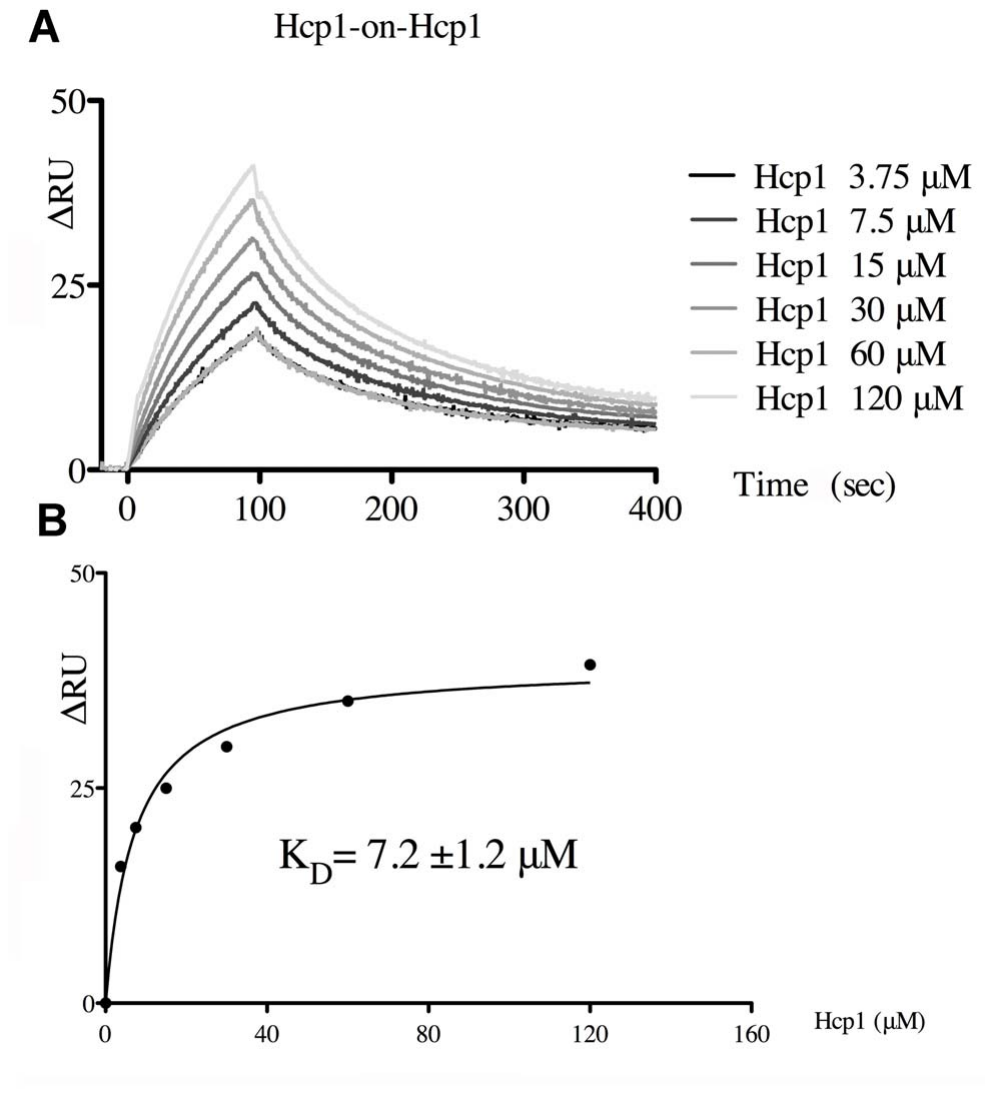
Interaction study of Hcp1/Hcp1 hexamers using surface plasmon resonance. (A) Binding pattern of Hcp1 (3.75 to 120 µM) on Hcp1 covalently immobilized on the CM5 chip. The variation of plasmon resonance is reported on the *y* axis (in arbitrary unit; ΔRU) and the reaction time on the *x* axis. (B) Graph representing the equilibrium response level (ΔRU; *y* axis) plotted as a function of the Hcp1 concentration (µM, *x* axis), with t curve fit to 1∶1 equilibrium model for determination of the K_D_ at 50% saturation.

All the Hcp proteins purified so far are hexameric in solution except for the EpvC protein of *E. tarda*, which was found in both dimeric and hexameric states in solution [18]. Furthermore, no *in vitro* self-association of the hexamers was reported to date, and only the presence of optimally-engineered Cys residues allowed to observe formation of tubular structures by transmission electron microscopy (TEM) [36]. However, despite the fact that this was never directly observed *in vitro*, Hcps should form tubes *in vivo* to allow the delivery of toxins into prey cells. Indeed, we recently evidenced tubular structures of Hcp *in vivo* using targeted disulfide bridges [10]. To gain insights into the stacking of Hcp1 hexamers, we investigated the self-association of Hcp1 using Surface Plasmon Resonance (SPR). To this end, Hcp1 hexamers were coupled to a CM5 SPR chip and Hcp1 was passed over the chip as analyte. We observed fast association and dissociation (Fig. 4A) between the bound and the circulating hexamers, with saturation occurring with Hcp1 concentrations above ∼50 µM (Fig. 4B). Analysis of the saturation curve yielded a K_D_ value of 7.2±1.2 µM. Both the k_off_ and K_D_ values are consistent with a fast exchange. This rapid exchange between Hcp hexamers explains why Hcp1 hexamers do not self-assemble to form tubes *in vitro*. We then examined by SPR the self-association of the Hcp1_WW_ hexamer, but we could not identify any interactions in the conditions used for the native Hcp.

### Oligomerization Studies of Hcp1_G96C/S158C_

**Figure 5:**
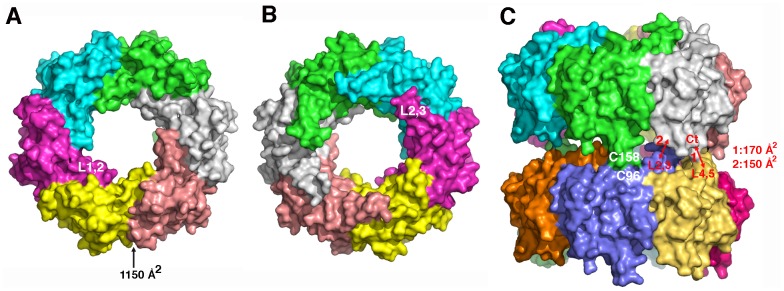
Hexamers of Hcp1_WW_ and model of the hexamers stacking in the Hcp1_G96C/S158C_ tube assembly. (A, B) Top- (A) and bottom- (B) view surface representations of Hcp1_WW_ hexamers. The positions of the two overhang L1,2 and L2,3 loops are indicated. The buried surface at monomer interface is indicated. (C) Surface representation of a two stacked hexamers model of Hcp1_G96C/S158C_. The cysteine residues implicated in disulfide bond formation are indicated, as well as the major determinants at the hexamer-hexamer interface with their surface buried areas.

Oligomerisation of Hcp1_WW_ in solution was investigated using MALLS-SEC-UV. The resulting chromatogram clearly indicated the presence of hexamers in solution (126 kDa), as with native Hcp1 (data not shown). In these hexamers, the X-ray structure indicates that each subunit surface exhibits an interface area of 1150 Å^2^ on each face, for a total monomer surface of 9000 Å^2^ (Fig. 5A,B). Each interface counts for 13% of the total surface (26% for both interfaces), explaining the strength of Hcp monomers association within the hexameric ring.

**Figure 6:**
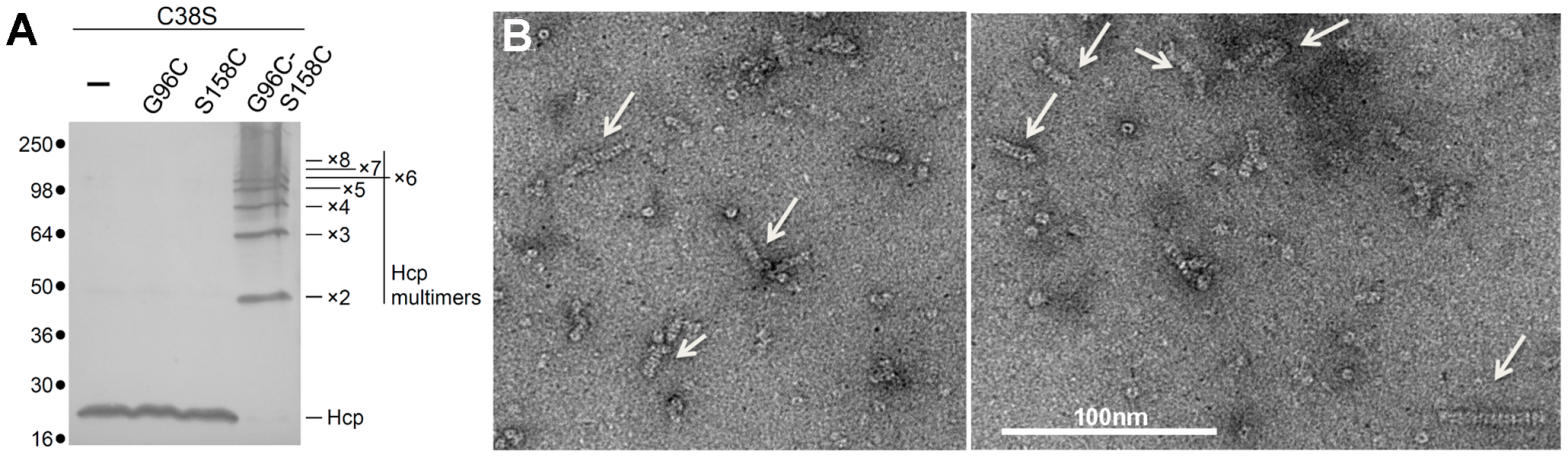
Hcp1_G96C/S158C_ tube formation as shown by *in vivo* disulfide bond formation and Electron Microscopy. (A) Cytoplasmic extracts from EAEC Δ*hcp1* cells producing the indicated cysteine Hcp1 mutant proteins after *in vivo* oxidative treatment with copper phenanthroline were loaded on a 12.5%-acrylamide SDS PAGE and immunodetected with the anti-FLAG monoclonal antibody. Positions of the Hcp1 monomer and multimers are indicated on the right. Molecular weight markers (in kDa) are indicated on the left. (B) Representative electron micrographs of negatively-stained Hcp1_G96C/S158C_ particles. Hcp1_G96C/S158C_ tubular structures are indicated by arrows. Scale bar, 100 nm.

The crystal structures of the Hcp proteins showed that Hcp hexamers can be arranged in a head-to-head, head-to-tail or tail-to-tail packing [1], [17], [18]. Therefore, we recently developed an assay to determine how Hcp hexamers are organized *in vivo*. For this, we engineered Hcp1 cysteine variants to induce disulfide bond formation between two hexamers [10]. This approach demonstrated that Hcp1 hexamers are stacked on each other in a head-to-tail conformation in the cell cytoplasm. As shown previously [10], once a cystein-less Hcp1 protein (C38S) bearing the G96C and S158C substitutions was produced in EAEC, bands corresponding to disulfide cross-linked oligomers (up to 8 monomers) can be visualized by SDS-PAGE (Fig. 6A). To confirm these data *in vitro*, the Hcp1_G96C/S158C_ double mutant protein was produced and purified by nickel affinity and gel filtration. The negative-staining electron micrographs revealed the presence of tubes constituted of up to ∼15 stacked hexamers (Fig. 6B).

Cys-96 and Cys-158 being able to form disulfide bonds in the Hcp1_G96C/S158C_ tubes, we modeled the hexamer/hexamer interaction using COOT [37] (Fig. 5C). The two positions of the substitutions, 96 and 158, are located in a mobile loop (L4,5) and at the C-terminus, respectively. Trp-158 is the last visible residue in our electron density map, and loop L4,5 is only visible in the electron density map of one monomer. Analysis of the model indicates that interactions occuring between hexamers cover only a total of 350 Å^2^ for each monomer. This interface surface area is only about one third of that found between monomers within the hexamer, and therefore explains (i) the low affinity measured between hexamers by SPR and (ii) the inability of Hcp1 to assemble tubes *in vitro*.

## Discussion

### Hcp1 Crystal Structure and Tube Assembly

In this study, we report the crystal structure of an Hcp1 variant and analyzed the mechanism of assembly of Hcp hexamers, the building blocks of the T6SS injection tube. First, we showed that Hcp1 displays a 3D structure comparable to those determined previously for other members of the Hcp family [1], [17], [18]. Recently, we showed that the Hcp1 protein from EAEC assembles tubular structures *in vivo* in a head-to-tail conformation [10]. By engineering mutations at strategic positions for tube assembly – Asn-93/Gly-96 and Ser-158– located in the overhang loop L4,5 (Ile91 to Gly96) and at the C-terminus, respectively, we provide a better understanding of Hcp hexamers stacking. First, insertion of bulky tryptophan residues at these positions to yield the Hcp1_WW_ protein abolished the biologically relevant stacking. Not only the Trp indole side-chains prevent tube elongation by head-to-tail hexamers stacking along a central axis, but they also promote a 1/2 head-to-head hexamers interaction by strong aromatic interactions with their symmetry related Trp side chains. The disruption of the *in vitro* packing is consistent with the observation that introduction of tryptophan residues at the same positions prevents Hcp1 tube formation *in vivo* [10]. By contrast, introduction of cysteine residues at the same positions stabilized Hcp1 tube formation through formation of disulfide bonds between hexamers and allowed to evidence Hcp tubes *in vivo* and visualize Hcp tubes by electron microscopy. This nanotubes are reminiscent of those observed with *P. aeruginosa* Hcp1 cystein variants [36]. The Hcp1_WW_ and Hcp1_G96C/S158C_ variant therefore appeared to be useful tools to study Hcp1 tube assembly both *in vivo* and *in vitro*.

### Commonalities and Diversities of T6SS and Bacteriophage Tail Assembly

Hcp1 hexamers, like other Hcp proteins studied to date are not able to form tubes in the absence of the other components of the T6SS machine [1], [11]. In phages, tail assembly is triggered by the Initiation Complex (IC), a complex composed of the baseplate and the tape measure protein (TMP), a long coil-coiled protein that determines the length of the phage tail. In the IC, the TMP is covered by chaperones [38], [39] which are progressively replaced by the major tail protein (MTP) that forms stacked hexamers helically disposed around the TMP. Tube polymerization is completed once the tail terminator protein recognizes the TMP’s end and caps the MTP tube. It has been proposed that MTP hexamer formation, and initiation of the tail tube polymerization probably occurs via conformational switching catalyzed by the IC and then by the properly folded MTP hexamers themselves [40]. In *Myoviridae*, the tail sheath wraps the MTP hexamers after the tube is completed [40]. Noteworthy, once formed, the tail tube structure is very robust and resists the ejection of TMP after infection. In *Siphoviridae*, while disconnection of baseplate and capsid from the tail is often observed, the tail is a tough device surviving most events. The T6SS tube therefore shares similarities and exhibits differences with the bacteriophage tail tube. First, no TMP homologue or equivalent has been identified in T6SS machines. However, it is clear that baseplate components such as VgrG and TssE, the T6SS counterparts of the gp27/gp5 hub complex and of the gp25 wedge subunit respectively, are necessary for proper assembly of the T6SS tail structure [8], [10], [15]. The complexity of the bacteriophage baseplate suggests that additional T6SS baseplate-like components remain to be identified. The structure of the T6SS and bacteriophage tubes and their conformational flexibilities probably exhibit significant differences as Hcp proteins are able to form extremely stable hexamers, a feature that is not shared by MTPs, that remain monomeric in solution [9] making conformational switching unlikely. However, these hexamers are loosely associated *in vitro* as shown by our SPR studies, whereas phages tail tubes are extremely stable.

By contrast to the T6SS tail tube Hcp proteins, the tail sheath composed of the TssB and TssC proteins encoded within the *sci1* or *sci2* T6SS gene clusters form long tubes as observed by TEM (B. Douzi and C. Cambillau, unpublished data), a result consistent with the tail sheaths produced by the *V. cholerae* [27] and the *P. aeruginosa* HSI-1 T6SS [29]. However, the TssBC tubular structures lack homogeneity since image reconstructions have demonstrated that they have 12- or 13- fold symmetry [29].

Regarding the T6SS assembly mechanism, the wealth of data gathered to date, as well as our results described in this manuscript, suggest a possible scheme. As suggested by fluorescence microscopy experiments [10], [30], the pre-formed Hcp hexamers might act as a template for TssBC tubes assembly with well-defined dimensions, most probably dodecamers. However, since Hcp is not able to form tubes by itself, Hcp hexamers stacking might be initiated once attached to the hub or when a baseplate-like structure, composed at least of TssE and VgrG, is assembled. These Hcps could be either alone or loaded with their specific effectors [24]. Recent data have shown that *in vivo* Hcp tube formation required the VgrG protein but is independent of the TssBC sheath proteins [10]. By contrast, polymerizing Hcps are required for sheath assembly. It is then possible to envisage two models. First, the first Hcp hexamer associated on the baseplate might serve as template for the recruitment and the association of a first TssBC ring of proper size (*e.g.*, a dodecamer) with would in turn serve as a scaffold for TssBC tube elongation. Then, polymerizations of the Hcp tube and of the TssBC sheath will be coupled and concomitant. In the second model, the tail tube will be first completed before serving as template for sheath assembly. This second model is consistent with the phage assembly process, in which the completed tail tube serves as template for the sheath [41]. However, in favor of the first model, the length of the T6SS Hcp tube does not seem to be controlled, and no signal for completion should exist hence hampering initiation of sheath polymerization. Further *in vivo* and *in vitro* evidence are required to discriminate these two models but the approaches and the tools developed recently will help to gain insights into this mechanism and to better compare T6SS and bacteriophage tail assembly.

## Materials and Methods

### Cloning and Site-directed Mutagenesis

The DNA sequences encoding the Hcp1 and Hcp2 proteins were amplified from chromosomal DNA of enteroaggregative *E. coli* 17-2 using specific primer pairs (Table S1), and cloned into the pDEST14 expression vector using standard Gateway protocols [42] to yield pDEST14-Hcp1 and pDEST14-Hcp2 respectively. These constructions led to the production of the full-length Hcp1 and Hcp2 proteins fused to a C-terminal 6×His tag.

QuickChange PCR-based targeted mutagenesis of the *hcp1* gene was performed using the pDEST14-Hcp1 and pUC-Hcp_FLAG_ [43] vectors as DNA templates and pairs of specific primers (listed in Table S1) bearing mismatches in the targeted codon to introduce the desired mutations. Mutations were confirmed by DNA sequencing (GATC biotech).

### Overproduction and Purification of the EAEC Hcp Proteins

Hcp1 and Hcp2 wild-type and mutant proteins were purified using an identical protocol. Briefly, *E. coli* BL21(DE3) pLys S (Invitrogen) cells were transformed with the pDEST-14 derivatives. Overnight cultures grown on Terrific Broth (TB; 1.2% peptone, 2.4% yeast extract, 72 mM K_2_HPO_4_, 17 mM KH_2_PO_4_, and 0.4% glycerol) supplemented with ampicillin (100 µg/ml) and chloramphenicol (35 µg/ml) at 37°C were diluted in TB medium and grown at 37°C to an OD_600_ = 0.6. The temperature was then decreased to 25°C and the expression of the *hcp* genes was induced by IPTG (500 µM) for 18 hours. Cells were harvested, resuspended in buffer A (50 mM Tris pH 8.0, 300 mM NaCl) supplemented with EDTA (1 mM), lysozyme (0.5 mg/ml), and phenylmethylsulfonyl fluoride (PMSF), submitted to three freeze-thawing cycles and sonicated after the addition of DNase (20 µg/ml) and MgCl_2_ (20 mM). Insoluble material was discarded by centrifugation for 30 min at 16000×*g*. All the subsequent purification steps were performed using an AKTA FPLC system. First, the soluble fraction was loaded into a 5-mL HisTrap FF colomn (GE Health Sciences). After extensive washing, the Hcp proteins were eluted in one step gradient of Imidazole 250 mM in Buffer A. The second purification step consisted to a gel filtration on a Sephadex 200 26/60 column (GE Health Sciences) in Tris 20 mM, NaCl 100 mM at pH 8.

### Biophysical Methods

Size exclusion chromatography (SEC) was performed on an Alliance 2695 HPLC system (Waters) using KW803 and KW804 columns (Shodex) with a Tris-HCl 20 mM (pH 7.5) NaCl 100 mM buffer, and a flow of 0.5 ml/min. Analysis using MALS, UV spectrophotometry, QUELS and RI were performed with a MiniDawn Treos (Wyatt Technology), a Photo Diode Array 2996 (Waters), a DynaPro (Wyatt Technology) and an Optilab rEX (Wyatt Technology), respectively, as previously described [44]. Mass and hydrodynamic radius were calculated with the ASTRA software (Wyatt Technology) using a *dn/dc* value of 0.180 mL/g.

### Crystallization, Data Collection, Processing and Refinement

The final concentration of the Hcp1_WW_ protein preparation was 28 mg/ml. Hcp1_WW_ crystallization trials were carried out by the sitting-drop vapor diffusion method in 96-well Greiner crystallization plates at 20°C using a nanodrop-dispensing robot (Cartesian Inc.). Crystals grew in 3 days after mixing 200 nl of Hcp1_WW_ at 28 mg/ml with 100 nl of PEG3350 (17%), Bis-Tris-Propane (15 mM), Magnesium Formate (0.1 M), pH 6.75. Crystals were cryoprotected with their mother liquor. A 1.69 Å resolution data set was collected at the ESRF beamline ID29 (Grenoble, France). The data set was processed using XDS [45], and scaling was performed using XSCALE [45] (Table 1). The structure of the 6×His Hcp1_WW_ protein was solved by molecular replacement using the structure of Hcp3 (PDB entry 3HE1) from *P. aeruginosa* as a model. Structure refinement was performed with AutoBUSTER [46] alternated with model rebuilding using COOT [37] (Table 1). Figures were made using PyMOL [47] or Chimera [48].

### Transmission Electron Microscopy

The protein samples were diluted to a final concentration of 0.02 mg/ml in Tris-HCl 50 mM pH 8, NaCl 100 mM before immobilization on a glow-discharged carbon grid by incubation for 1 minute. The particles were negatively stained with uranyl formate. Grids were air-dried and electron micrographs were collected using a FEI Tecnai 12G2 Spirit microscope operated at 120 kV with a 60 K magnification.

### Interaction Studies using Surface Plasmon Resonance

Steady state of the interaction between Hcp1 hexamers was performed using a BIAcore T200 at 25°C. A HC200 m (Xantech) sensor chip was coated with Hcp1, immobilized by amine coupling (ΔRU = 4300). A flow-cell was coated with a control ligand (Thioredoxin) immobilized by amine coupling at the same level of Hcp1 (ΔRU = 4100). Solutions of Hcp1 (3.75–120 µM) in HBS-EP buffer (Hepes 10 mM, NaCl 150 mM, EDTA 3 mM, T20 0.005%) were passed over the Hcp1-coated and control flow-cells. Binding traces were recorded in duplicate for 6 concentrations of Hcp1. The signal from the control flow cell and the buffer response were subtracted from all measurements. The k_off_ and K_D_ values were obtained using the fitting tool of the BIAevaluation software (BIAcore).

#### *In vivo* disulfide bond formation assay

A total of 3×10^10^ exponential growing Δ*hcp1* cells (OD∼0.6) producing the indicated Hcp cysteine variant were incubated with 0.3 mM dichloro(1,10-phenanthro- line) copper(II) (Cu-oP) for 20 min without agitation. Cells were then harvested by centrifugation and incubated in 10 mM HEPES (pH 7.4), Sucrose 30%, 1 mM EDTA and 2.5 mM N-ethyl-maleimide (NEM) for 30 min on ice to block free thiol groups. Cells were pelleted by centrifugation and the cytoplasm fraction was recovered by fractionation [43], mixed with loading buffer prior to analysis by SDS-PAGE and immunoblotting.

#### Accession codes

The atomic coordinates and structure factors have been deposited at the Protein Data Bank under accession code 4HKH.

## Supporting Information

Table S1 **Primers used for the EAEC Hcp1/Hcp2 amplification step Oligonucleotides used for site-directed mutagenesis of EAEC Hcp1.**
(DOCX)
