# Supplementary material for: Crystal Structure and Self-Interaction of the Type VI Secretion Tail-Tube Protein from Enteroaggregative Escherichia coli
Source: PLoS One. 2014 Feb 14;9(2):e86918. doi: 10.1371/journal.pone.0086918 (PMC3925092; doi:10.1371/journal.pone.0086918)
Supplement: Table S1 — Primers used for the EAEC Hcp1/Hcp2 amplification step Oligonucleotides used for site-directed mutagenesis of EAEC Hcp1. (DOCX) [file pone.0086918.s001.docx]

**Supplemental Table S1**

**Primers used for the EAEC Hcp1/Hcp2 amplification step**

**Oligonucleotides used for site-directed mutagenesis of EAEC Hcp1**

- Hcp1 primers :

Forward : ^5’^GGGGACAAGTTTGTACAAAAAAGCAGGCTTAgaaggagatagaaccATGAAAGCAATTCCAGTTTATCTGTG^3’^

Reverse : ^5’^GGGGACCACTTTGTACAAGAAAGCTGGGTTTATTA ATGGTGATGGTGATGGTG CGCGGTGGTACGCTCACTCC^3’^

- Hcp2 primers :

Forward : ^5’^GGGGACAAGTTTGTACAAAAAAGCAGGCTTAgaaggagatagaaccATGAAAGTCGGAGTTATGAGTAATTC^3’^

Reverse : ^5’^GGGGACCACTTTGTACAAGAAAGCTGGGTTTATTAATGGTGATGGTGATGGTGTACAAGAGCCTCTTTATATAAG^3’^

- Amino-acid substitutions (to tryptophane) ^*^

N-93

GTTTAAGTGGTACAAAATCTGGGATGCCGGTCAGGAGGTG

Ser-158

CATTCATTCCGACGCATGGTGGGAGCGTACCACCGCGTAA

- Amino-acid substitutions (to Cystein) ^*^

Gly-96

GTACAAAATCAATGATGCCTGTCAGGAGGTGGAGTATTT

Ser-158

CATTCATTCCGACGCATGGTGTGAGCGTACCACCGCGTAA

* Mutagenesized codon underlined.
